# Association Between Tibiofemoral Bone Shape Features and Retears After Anterior Cruciate Ligament Reconstruction

**DOI:** 10.1177/23259671241289096

**Published:** 2024-11-19

**Authors:** Karsyn N. Bailey, Kenneth T. Gao, Ryan T. Halvorson, Jacob F. Oeding, Sharmila Majumdar, Valentina Pedoia, Drew A. Lansdown

**Affiliations:** * Department of Orthopaedic Surgery, Washington University in St Louis, St Louis, Missouri, USA; † University of California, Berkeley–University of California, San Francisco Graduate Program in Bioengineering, San Francisco, California, USA; ‡ Center for Intelligent Imaging, Department of Radiology, University of California, San Francisco, San Francisco, California, USA; § Department of Orthopaedic Surgery, University of California, San Francisco, San Francisco, California, USA; ‖ Mayo Clinic Alix School of Medicine, Rochester, Minnesota, USA; ¶ Altos Labs, Los Altos, California, USA; Investigation performed at the University of California, San Francisco, San Francisco, California, USA

**Keywords:** knee, ACL, magnetic resonance imaging, radiology, injury prevention

## Abstract

**Background:**

A retear after anterior cruciate ligament (ACL) reconstruction remains a common and devastating complication. Knee bone morphology is associated with the risk of ACL injuries, ACL retears, and osteoarthritis, and a combination of tools that derive bone shape from clinical imaging, such as magnetic resonance imaging (MRI) and statistical shape modeling, could identify patients at risk of developing these joint conditions.

**Purpose:**

To identify bone shape features before primary ACL reconstruction in patients with an eventual retear compared to those with a known intact ACL graft.

**Study Design:**

Case-control study; Level of evidence, 3.

**Methods:**

Bone was automatically segmented on 2-dimensional proton density–weighted MRI of the knee in patients at the time of the initial ACL injury using deep convolutional neural networks. Patients with a subsequent retear after reconstruction within 3 years (22 femurs, 19 tibias) were compared with those with an intact ACL graft at 3 years (20 femurs, 22 tibias) using statistical shape modeling to identify preoperative bone shape features predictive of a retear after ACL reconstruction.

**Results:**

Statistical shape modeling revealed 2 specific bone shape features (modes) in the femur and 1 mode in the tibia that demonstrated significant differences at the time of the initial injury in patients with subsequent retears. In the femur, a narrower intercondylar notch width, a widened medial condylar width, an increased femoral condylar offset ratio, increased surface area along the lateral femoral condyle relative to the medial condyle, and a more prominent trochlear sulcus at the time of the initial injury were associated with retears after ACL reconstruction. In the tibia, a diminished ACL facet prominence, a squared lateral and medial tibial plateaus, and a broader and flattened tibial spine at the time of the initial injury were associated with retears after ACL reconstruction.

**Conclusion:**

Using the automatic bone segmentation pipeline on preoperative MRI, the authors identified bone shape features associated with a retear after ACL reconstruction. The use of this pipeline enables large-scale studies of bone shape on MRI and could predict patients at risk of ACL retears to alter treatment decisions.

Knee bone morphology has been recognized as a key risk factor for anterior cruciate ligament (ACL) injuries, negative outcomes after ACL reconstruction, and the development of knee osteoarthritis. In particular, notch morphology,^3,29^ tibial slope,^3,10^ tibial plateau width,^10^ femoral condyle characteristics,^10^ and trochlear inclination^31^ have been shown to increase the ACL injury risk and negatively impact postoperative outcomes, including persistent instability, the retear risk, patient-reported outcomes, and cartilage degeneration.^5,7,20^ Other studies have employed statistical shape modeling to identify patterns of 3-dimensional (3D) shape variations associated with postoperative outcomes beyond what is typically captured with simple geometric measurements.^2,24,31^ Understanding pertinent morphological risk factors can better inform the surgical technique and postoperative care for patients with ACL injuries.

Rates of retears after ACL reconstruction remain notably elevated, reaching up to 40% in certain cohorts.^16^ Several identifiable factors, including hyperlaxity, young age, and an early return to cutting or pivoting activities, are established risk factors for a retear. In addition to rehabilitation strategies to improve stability, emerging research has centered around surgical interventions at the time of initial reconstruction, such as lateral extra-articular tenodesis (LET), anterolateral ligament reconstruction, and corrective osteotomy, as well as the selection of graft type, to reduce the risk of retears after reconstruction .^13-16,18,21^ The improved identification of patients at the highest risk of graft failure could potentially pave the way for more targeted interventions at the time of the initial injury.

As prior research has largely focused on smaller clinical cohorts, there is a substantial need for higher powered studies using large cohorts to identify precise population parameters for these morphological risk factors.^22^ Although computed tomography (CT) is the gold standard for assessing bone morphology, similar accuracy in deriving tibiofemoral bone shape has been achieved using magnetic resonance imaging (MRI).^19,28^ However, those studies relied on either labor-intensive manual segmentation or high-resolution MRI sequences, typically with a 1-mm slice thickness and often nearly isotropic to enhance the collection of sagittal-plane information. The increased time involved with manual segmentation or increased acquisition times with high-resolution MRI limit the feasibility for larger scale analyses in the clinical setting.

The development of automated tools to assess bone shape on clinical-grade MRI sequences could allow for analysis of large datasets necessary for higher powered studies. Machine learning has previously been used to develop automated bone segmentation tools from high-resolution MRI to uncover key morphological features of tibiofemoral bone shape.^4,8,12,19^ We aimed to use machine learning to develop an automated pipeline that segments knee bone morphology from clinical sequences of proton density (PD)–weighted MRI for bone shape analysis. Given the abundance of clinical PD-weighted MRI sequences, the application of this pipeline could enable further large-scale studies of key bone shape features predictive of a variety of clinical outcomes.

The purpose of this study was to validate our automated MRI-based segmentation pipeline against manually segmented CT and employ it to derive knee bone morphology from preoperative clinical MRI in patients with ACL injuries. We hypothesized that our automated pipeline would identify predictive bone shape features in the tibiofemoral joint associated with a retear after ACL reconstruction. We further hypothesized that the use of this pipeline clinically would detect patients at risk of ACL retears, impact surgical treatment decisions, and allow for large-scale studies on MRI to uncover shape differences in a variety of joint injuries.

## Methods

### Participants

We retrospectively reviewed operative records from our tertiary university-based sports medicine practice at the University of California, San Francisco, between 2009 and 2017 to identify patients undergoing revision ACL reconstruction. We then included any patient who had undergone primary reconstruction with our group to control known factors for subsequent ACL reconstruction failure. The tunnel position was evaluated and confirmed to be appropriately placed on postoperative radiographs. The group with eventual ACL retears comprised patients in whom no clear factor was identified for failure of initial reconstruction, including graft diameter, a missed ligamentous injury, or a meniscus-deficient knee from prior subtotal or total meniscectomy. Patients were required to have a 2-dimensional (2D) PD-weighted sagittal MRI sequence (3-mm slice thickness) available in our picture archiving and communication system and completed on a 3-T scanner.

The comparative group was composed of patients with a known intact ACL graft at 3 years after primary ACL reconstruction, selected to control for sex, body mass index, and age at the time of the initial injury. All patients in the comparative group had undergone 2D PD-weighted sagittal MRI preoperatively.

Overall, we included 48 patients with a retear within 3 years after ACL reconstruction and 44 patients with a known intact ACL graft at 3 years after ACL reconstruction. Because of imaging limitations, such as poor image quality, poor segmentation due to varying acquisition parameters, and poor alignment using the iterative closest point algorithm, there were 20 patients without subsequent retears and 22 patients with subsequent retears for femur analysis as well as 22 without subsequent retears and 19 with subsequent retears for tibia analysis. The study protocol was approved by the institutional review board.

### Development and Testing of Deep Learning Algorithm

To develop the deep learning algorithm, MRI scans of 68 patients who had clinical PD-weighted (3.5-mm slice thickness) sagittal MRI sequences of the knee were obtained. An automatic femur and tibia segmentation framework was developed by training 2 deep convolutional neural networks using 2D PD-weighted MRI sequences (3.5-mm slice thickness) in patients with ACL tears. Pairs of contralateral and ipsilateral (injured) knees from 68 patients were utilized. The femur and tibia were manually segmented using custom MATLAB-based software (MathWorks) and randomly split into 28 training, 6 validation, and 6 test sets. Both preoperative and postoperative reconstructions were included in the training and validation datasets (22 preoperative, 18 postoperative). Additionally, two 2D V-Net models^23^ were independently trained. The model inputs (ie, MRI scans) were minimum-maximum (0, 1) normalized volumetrically, and the corresponding segmentation masks were represented as one-hot–encoded multiclass arrays, with classes as pixels representing the femur, tibia, and patella. Model training was performed with the Adam optimizer and soft Dice loss following the softmax function of the final logits. Training was terminated with an early stopping rule, and the model checkpoint with the highest Dice similarity coefficient on the validation loss was selected for inference. Final volumetric segmentations on all MRI scans were assembled from the respective best model outputs. The volumetric Dice similarity coefficient, measured in the unseen test sets, was used to assess segmentation performance.

### Comparison of Automatically Segmented MRI to Manually Segmented CT

CT is often the gold standard for evaluating bone morphology because of its high delineation of bone and small pixel spacing and therefore was utilized to compare the precision of bone derived from automatic segmentation of MRI to that derived from manually segmented CT. A distinct group of patients from those described earlier who had both 2D PD-weighted MRI sequences (3-mm slice thickness) and ipsilateral knee CT scans (0.625-mm slice thickness) were identified (9 femurs, 9 tibias). In this group of patients, the femur and tibia were automatically segmented on MRI scans using the automatic segmentation pipeline, and masks outside the range of initial and final slices were manually removed. On CT scans, the femur and tibia were manually segmented using custom MATLAB-based software. Then, 3D triangulated meshes of automatically segmented MRI scans and manually segmented CT scans were produced using the marching cubes algorithm, and the number of faces was reduced and normalized across all scans. Vertices of the triangulated meshes of each MRI scan were aligned and matched to those of the respective CT scan for each patient using the iterative closest point algorithm. Vertex-to-vertex distances between MRI and CT were measured to estimate the degree of segmentation error propagated to the 3D surface meshes. The Dice similarity coefficient (DSC) was used to evaluate segmentation performance, with DSC values >70% considered excellent overlap for image validation.^32,33^ Common features associated with an ACL tear, including notch width, femoral condylar width, lateral femoral condylar ratio, and tibial plateau width, were manually measured on the 3D triangulated meshes generated from MRI and CT, and the mean difference in distances between MRI and CT was calculated.

### Statistical Shape Modeling of Knee Bone Morphology

To demonstrate the feasibility of applying the automatic segmentation pipeline to clinical questions, patients with ACL tears who had 2D PD-weighted MRI sequences (3-mm slice thickness) at the time of the initial injury were identified. These patients represent an independent subgroup of patients as those described earlier. Again, 3D triangulated meshes were generated, as above, scaled to a reference based on the anterior-to-posterior distance and aligned and matched to an atlas using the iterative closest point algorithm. Modes of shape variance were determined using principal component analysis (PCA). Each mode, ordered by the amount of geometric variance, described a unique shape feature orthogonal to all other modes in the PCA space. Unlike simple measurements (notch width, condylar width, etc), a mode in statistical shape modeling can include a more complex shape feature. The effect of each mode was modeled by 3D surfaces for each specific shape feature. The modes were visualized at 3 standard deviations above and below the mean to capture specific changes in the femur and tibia. Modes were considered statistically significant if the mean PCA score between groups was significant, indicated by *P* < .05 using the Student *t* test. Statistics were done in Matlab 2015b (MathWorks).

## Results

### Bone Morphology on Automatically Segmented MRI Is Clinically Equivalent to That on Manually Segmented CT

**Figure 1.**
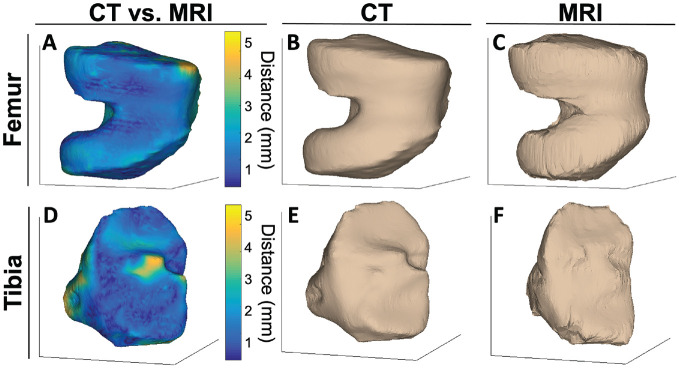
Vertex-to-vertex distances between computed tomography (CT) and magnetic resonance imaging (MRI) illustrated using a colormap of 3-dimensional triangulated meshes from a representative patient were homogeneous across the bone (femur [A], tibia [D]). Qualitative comparison of 3-dimensional triangulated meshes generated from manually segmented CT (femur [B], tibia [E]) and automatically segmented MRI (femur [C], tibia [F]) demonstrated comparable bone morphology for the ipsilateral knee of a representative patient.

Compared with manual segmentation of CT, segmentation performance of the test sets from MRI had a DSC of 95.29% (95% CI, 94.85%-95.73%) for the femur and 95.52% (95% CI, 94.77%-96.27%) for the tibia. An evaluation of vertex-to-vertex differences between MRI and CT measured with 3D triangulated meshes showed excellent agreement across all 9 femurs and tibias, with a mean vertex-to-vertex distance of 2.09 mm (95% CI, 1.72-2.46 mm) for femurs and 2.46 mm (95% CI, 1.93-2.99 mm) for tibias. Representative images of colormap-illustrated differences of the femoral condyle (Figure 1A) and proximal tibia (Figure 1D) showed qualitatively homogeneous distances between CT and MRI. The 3D triangulated meshes generated from CT (Figure 1, B and E) and MRI (Figure 1, C and F) were comparable and relatively homogeneous across the bone. Mean differences and percentage differences between CT and MRI for intercondylar notch width, lateral condylar width, medial condylar width, lateral femoral condylar ratio, lateral tibial plateau width, medial tibial plateau width, and total tibial plateau width are shown in Table 1.

**Table 1:** Mean Differences Between Manually Segmented CT and Automatically Segmented MRI^*a*^

	Mean Difference	Upper Limit of Agreement	Lower Limit of Agreement	Percentage Difference, %
Intercondylar notch width, mm	0.56 (–0.77 to 1.89)	3.96 (1.65 to 6.27)	−2.84 (–5.15 to −0.53)	3.13
Lateral condylar width, mm	0.58 (–0.55 to 1.70)	3.45 (1.50 to 5.40)	−2.29 (–4.24 to −0.34)	2.10
Medial condylar width, mm	0.16 (–1.01 to 1.33)	3.14 (1.12 to 5.16)	−2.82 (–4.84 to −0.80)	0.58
Lateral femoral condylar ratio	−0.022 (–0.044 to 0.0004)	0.034 (–0.004 to 0.072)	−0.078 (–0.120 to −0.040)	3.79
Lateral tibial plateau width, mm	−0.54 (–1.97 to 0.90)	3.12 (0.64 to 5.61)	−4.20 (–6.69 to −1.71)	2.05
Medial tibial plateau width, mm	−0.22 (–2.41 to 1.97)	5.37 (1.58 to 9.17)	−5.80 (–9.60 to −2.01)	0.89
Total tibial plateau width, mm	−1.18 (–3.79 to −1.43)	5.47 (0.96 to 9.99)	−7.83 (–12.35 to −3.31)	1.76

a The 95% CI is presented in parentheses . CT, computed tomography; MRI, magnetic resonance imaging.

### Shape Features in Femoral Condyles Predict Retears

**Figure 2.**
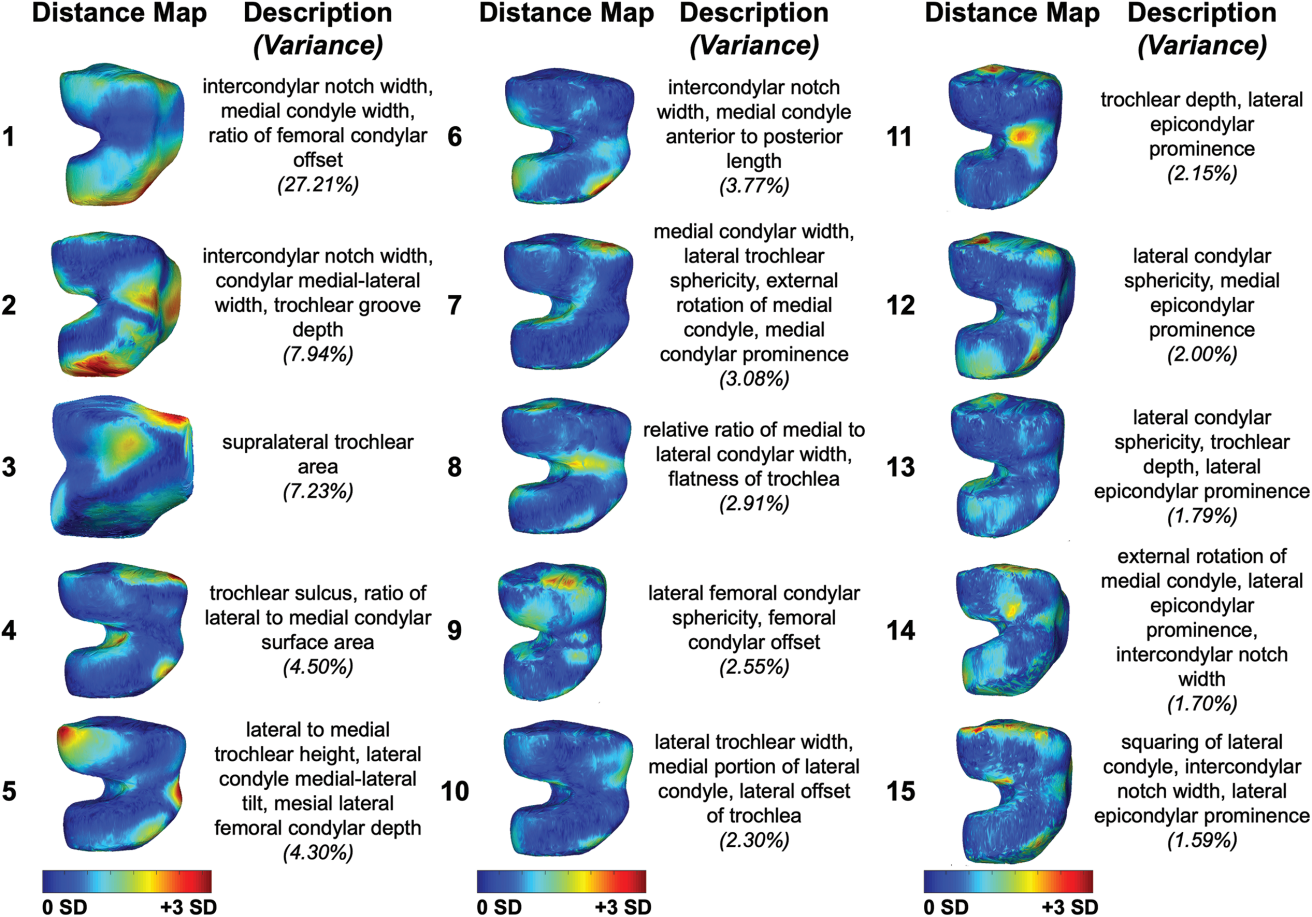
The first 15 principal component analysis (PCA) modes of the distal femur accounted for 75.04% of the PCA-normalized accumulated total variance. Each mode is represented by the spatial distribution of the vertex displacements equal to mean ± 3 SD, each oriented to illustrate the prominent features captured by the respective mode. Descriptions denote key shape features described by each mode. Variances represent the variance accounted for by each distinct mode.

**Figure 3.**
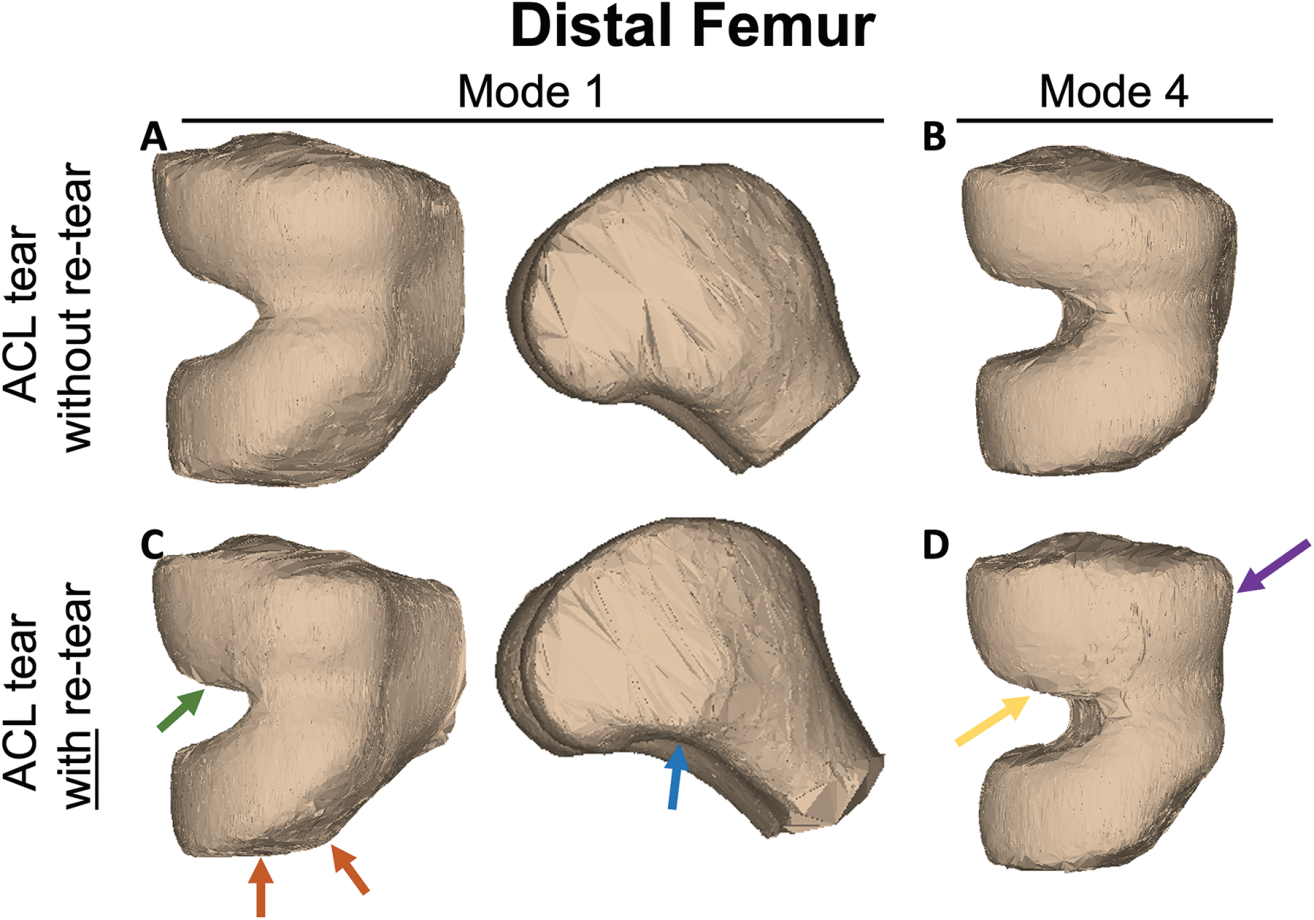
Statistical shape modeling of the femoral condyles with 15 principal component analysis modes revealed that modes 1 and 4 were significantly different (*P* < .05) at the time of the initial injury in patients who had subsequent anterior cruciate ligament (ACL) retears compared with those without retears. Images represent mean ± 3 SD from the average bone shape for each group. In patients with subsequent ACL retears, mode 1 illustrated narrowing of the intercondylar notch (green arrow), widening of the medial condyle (orange arrows), and an increased femoral condylar offset ratio at the time of the initial injury (blue arrow) (ACL tear without retear [A], ACL tear with retear [C]). Mode 4 showed an increase in the trochlear sulcus (purple arrow) and in the lateral femoral condylar surface area relative to the medial femoral condylar surface area (yellow arrow) at the time of the initial injury in patients with subsequent ACL retears (ACL tear without retear [B], ACL tear with retear [D]). Arrows denote spatial regions with notable shape differences.

The characteristics of patients with ACL tears are shown in Table 2. Femoral condylar bone morphological features within the dataset were illustrated by the first 15 modes of variation in bone shape derived from PCA (Figure 2), with *P* values of each PCA mode presented in Table 3. Comparison of morphology of the distal femur in those with subsequent retears after ACL reconstruction to those without retears using statistical shape modeling with 15 PCA modes revealed presurgical shape features associated with a retear. Modes 1 and 4 were significantly different (*P* < .05) at the time of the initial injury in patients who had subsequent retears after ACL reconstruction compared with those without retears. Femur mode 1 demonstrated a narrower intercondylar notch width, widened medial condylar width, and increased femoral condylar offset ratio at the time of the initial injury in patients with subsequent ACL retears (Figure 3, A and C). Femur mode 4 illustrated a more prominent trochlear sulcus and an increased surface area along the lateral femoral condyle relative to the medial condyle at the time of the initial injury in patients with subsequent ACL retears (Figure 3, B and D). The first 15 PCA modes of the distal femur accounted for 75.04% of the PCA-normalized accumulated total variance.

**Table 2:** Characteristics of Patients^*a*^

	Total	Intact Graft	Retear
Sex, %			
Female	47.2	44.4	50.0
Male	52.8	55.6	50.0
Body mass index, kg/m^2^	24.3 ± 3.4	24.3 ± 2.8	24.2 ± 4.2
Age at initial injury, y	28.0 ± 10.8	29.9 ± 9.0	25.9 ± 12.2
Age at revision, y	N/A	N/A	28.3 ± 12.1
Time to retear, y	N/A	N/A	2.4 ± 1.8
Graft diameter, mm	8.57 ± 0.77	8.54 ± 0.76	8.59 ± 0.80

a Data are presented as mean ± SD unless otherwise indicated. N/A, not applicable.

**Table 3:** *P* Values of Principal Component Analysis Modes^*a*^

Mode	Femur	Tibia
1	**.02773073**	.98976101
2	.15998297	.46505613
3	.90252542	.58165999
4	**.01815262**	.05373583
5	.51482353	.41043322
6	.87487949	.9884412
7	.7660945	**.02049406**
8	.41523515	.19586148
9	.8451149	.90534136
10	.2570405	.06671828
11	.70104968	.65720324
12	.09736011	.77507623
13	.2443013	.43303037
14	.14003843	.89405654
15	.20764734	.08109071

a The bolded values indicate *p* < 0.05.

### Shape Features in Tibial Plateau Predict Retears

**Figure 4.**
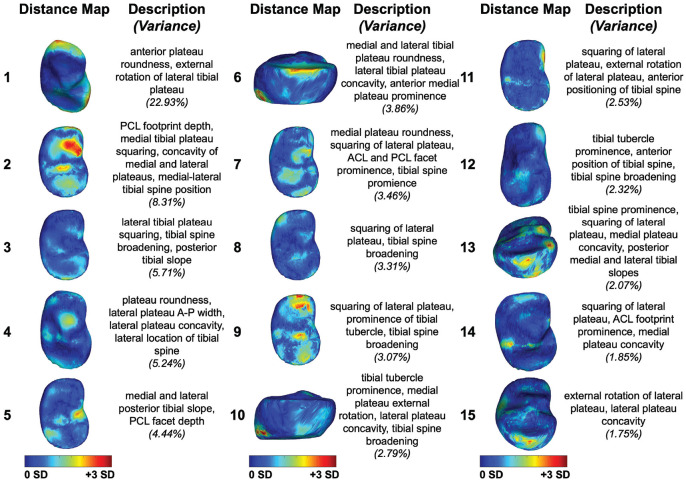
The first 15 principal component analysis (PCA) modes of the proximal tibia accounted for 73.60% of the PCA-normalized accumulated total variance. Each mode is represented by the spatial distribution of the vertex displacements equal to mean ± 3 SD, each oriented to illustrate the prominent features captured by the respective mode. Descriptions denote key shape features described by each mode. Variances represent the variance accounted for by each distinct mode. ACL, anterior cruciate ligament; A-P, anterior-to-posterior; PCL, posterior cruciate ligament.

**Figure 5.**
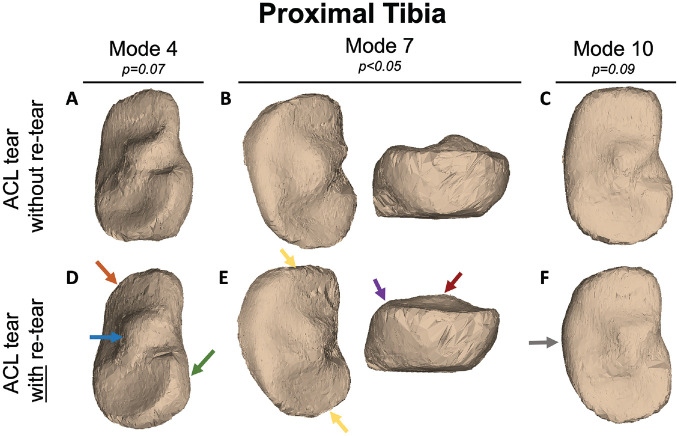
Statistical shape modeling of the tibial plateau with 15 principal component analysis modes revealed that mode 7 was significantly different (*P* < .05) at the time of the initial injury in patients who had subsequent anterior cruciate ligament (ACL) retears compared with those without retears. Modes 4 and 10 showed meaningful differences in patients with subsequent ACL retears (*P* = .05 and *P* = .06, respectively ). Images represent mean ± 3 SD from the average bone shape for each group. Mode 4 showed differences in the tibial plateau roundness (green arrow), an increased anterior-to-posterior width and convexity of the lateral tibial plateau (orange arrow), and a lateralized tibial spine (blue arrow) at the time of the initial injury in patients with subsequent ACL retears (ACL tear without retear [A], ACL tear with retear [D]). In patients with subsequent ACL retears, mode 7 illustrated a diminished ACL facet prominence (purple arrow), squared lateral and medial tibial plateaus (yellow arrows), and a broader and flattened tibial spine at the time of the initial injury (red arrow) (ACL tear without retear [B], ACL tear with retear [E]). Mode 10 showed increased external rotation of the medial tibial plateau (gray arrow), decreased concavity of the lateral tibial plateau, and broadening of the tibial spine at the time of the initial injury in patients with subsequent ACL retears (ACL tear without retear [C], ACL tear with retear [F]). Arrows denote spatial regions with notable shape differences.

PCA of the first 15 modes of variation in bone shape in the tibial plateau are shown in Figure 4, exhibiting the shape features identified at the time of the initial injury across the dataset. Statistical shape modeling of the tibial plateau with 15 PCA modes revealed that mode 7 was significantly different (*P* < .05) at the time of the initial injury in patients who had subsequent retears after ACL reconstruction compared with those without retears (Figure 5, B and E). Tibia mode 7 showed a diminished ACL facet prominence, squared lateral and medial tibial plateaus, and a broader and flattened tibial spine at the time of the initial injury in patients with subsequent ACL retears. Modes 4 and 10 in the tibia showed meaningful differences in patients with subsequent ACL retears (*P* = .05 and *P* = .06, respectively). Also, 3D reconstruction of tibia mode 4 captured differences in tibial plateau roundness, an increased anterior-to-posterior width of the lateral tibial plateau, increased convexity of the lateral tibial plateau, and a lateralized tibial spine at the time of the initial injury in patients with subsequent ACL retears (Figure 5, A and D). Tibia mode 10 showed increased external rotation of the medial tibial plateau, decreased concavity of the lateral tibial plateau, and broadening of the tibial spine at the time of the initial injury in patients with subsequent ACL retears (Figure 5, C and F). The first 15 PCA modes of the proximal tibia accounted for 73.60% of the PCA-normalized accumulated total variance.

## Discussion

Using an automatic bone segmentation pipeline on preoperative MRI, we identified shape features in the tibiofemoral joint present at the time of the initial injury that were associated with a subsequent retear after ACL reconstruction. Patients with subsequent ACL retears exhibited a variety of morphological differences at the time of the initial injury, captured by statistical shape modeling, including a decreased intercondylar notch width, a widened medial condylar width, increased convexity of the lateral tibial plateau, and a broadened and lateralized tibial spine. We employed an innovative deep learning approach to automatically segment PD-weighted MRI sequences for use in statistical shape modeling. Automatic segmentation of clinical MRI sequences demonstrated comparable bone morphology to 3D triangulated meshes generated from manual segmentation of CT scans for various clinically meaningful measurements, within the resolution necessary to detect relevant differences. Uncovering differences in knee bone morphology could yield predictive information for joint conditions and therefore alter treatment decisions to improve patient-specific decision-making.

Statistical shape modeling enables visual representation of 3D morphological patterns that capture differences otherwise unaccounted for by traditional 2D measurements. The application of this automatic segmentation pipeline to MRI identified distinct modes in the tibiofemoral joint that were associated with a retear after ACL reconstruction. Many morphological features in the tibiofemoral joint are known to be associated with an ACL injury, such as intercondylar notch width, femoral condylar offset ratio, and medial and lateral tibial slopes,^5^ and our analysis likewise identified these shape features. However, these measurements are typically limited to 2 dimensions, which restrict the ability to understand the complex 3D relationships among these features. Prior studies that utilized statistical shape modeling have shown similar predictive morphologies, but they derived morphological information from CT,^25^ required extensive manual segmentation of MRI,^9^ or used high-resolution MRI for automatic segmentation.^24^ Our validation work presented here showed comparable measurements between MRI and CT. Vertex-to-vertex measurements between MRI and CT in this study were relatively homogeneous as visualized on colormaps in Figure 1, the mean vertex-to-vertex measurement for the meshes was less than the slice thickness of MRI, and the percentage differences between MRI and CT for each measurement in Table 1 were within the range to detect meaningful differences in bone shape features. While CT offers improved bone resolution, MRI is most commonly performed in the setting of possible ACL injuries and before ACL reconstruction. This study employed an automatic segmentation pipeline on PD-weighted MRI, which has broad application for use in the clinical setting.

Statistical shape modeling provides key information about concurrent morphological patterns across the whole joint, offering more specificity for the overall joint phenotype that may predispose patients to an ACL injury. In certain modes, such as in femur mode 7 or femur mode 14, there was concurrent narrowing of the intercondylar notch width, accompanied by varying rotational alignment of the condyles to the diaphysis, rather than intercondylar notch narrowing strictly within the medial-to-lateral plane. Interestingly, these modes did not exhibit an association with a retear. A previous study has shown that in specific contexts, a reduction in intercondylar notch width is linked to an increased risk of ACL tears.^27^ However, in other contexts, intercondylar notch narrowing is not a risk factor for ACL retears.^1,11^ Our research suggests that intercondylar notch width in the context of the overall 3D joint shape may provide an explanation for this apparent contradiction. Furthermore, these findings underscore the importance of identifying 3D shape patterns that are associated with a retear to better identify patients at risk of a retear after ACL reconstruction.

This pipeline could be used clinically to identify one of the factors that place patients at risk of a retear after ACL reconstruction and allow for personalized treatment decisions tailored to a patient's individual tibiofemoral bone morphology. Operative treatment options for patients with ACL injuries, such as graft selection, LET, or osteotomy, can reduce the inherent risk of retears after ACL reconstruction. For example, LET in conjunction with ACL reconstruction decreases the rate of retears after ACL reconstruction.^13,15,16^ Furthermore, bone morphology can impact the efficacy of LET.^26^ The use of this automatic segmentation pipeline has the potential to screen for patients who would most benefit from personalized adjunctive operative decisions with ACL reconstruction that may reduce their risk of retears.

### Limitations

This study has several limitations. The PD-weighted MRI sequences used in this study had overall lower resolution relative to those in prior studies and thus may be underpowered to detect all shape features. However, this approach effectively identified clinically meaningful shape differences, even with lower resolution MRI, which highlights the robustness of the model. This automatic segmentation pipeline exhibited inconsistent accuracy in extracting bone from patients with significant synovial inflammation, and therefore, further work is needed to better optimize the model for patients with varying synovial inflammation. Likewise, segmentation performance dropped near the image periphery because of decreased signaling, which may impact shape differences in the femoral and tibial shafts. Conversely, this indicates higher quality measurements of shape features in the more relevant articulating regions of the joint. Patients with ACL tears were not included in the comparison between automatic segmentation of MRI and manually segmented CT. Because patients were selected based on the availability of both MRI and CT scans of the ipsilateral knee and those with hardware or other significant anatomic abnormalities were excluded, we do not expect this to dramatically change the ability of the model to detect shape features at a clinically meaningful resolution. Our work combined male and female patients and identified shape features predictive of morphological differences, independent of sex. Additionally, segmentation performance limitations restricted this project to a smaller sample size. Given the known sex-specific differences in bone shape and ACL injuries,^6,17,30^ future work could apply this pipeline to a larger sex-stratified population. We did not control for other variables that are known to increase the rate of failure, such as hyperlaxity. Bone shape, as identified here, is not the only risk factor for ACL retears; however, the findings, along with prior work in this area, do suggest that these identified features are an important consideration in identifying patients at risk of ACL retears.

Several patients were initially selected for inclusion in the study; however, imaging challenges, such as inconsistent accuracy in bone extraction from those with significant synovial inflammation and reduced signal clarity at the image periphery, reduced the number of 3D meshes that met the quality criteria for inclusion. To enhance the accuracy of our model and thus increase the pipeline throughput, future initiatives could involve integrating data from patients with substantial synovial inflammation into the training sets. Moreover, training the model using both high- and low-resolution MRI in the same patient would likely enhance the segmentation's precision and resolution at the image periphery.

## Conclusion

In this study, we utilized an automatic deep learning–based pipeline for statistical shape modeling of tibiofemoral bone morphology, demonstrating excellent agreement in 3D reconstruction and clinically meaningful measurements compared to the gold standard for bone segmentation: that is, CT. The application of this approach to preoperative MRI unveiled distinct bone morphological features present at the time of the initial injury associated with a retear after ACL reconstruction. In the femur, some of these morphological features included narrowing of the intercondylar notch width, widening of the medial condyle, an increased femoral condylar offset ratio, increased surface area along the lateral femoral condyle relative to the medial condyle, and an increased trochlear sulcus. In the tibia, some of these morphological features included a diminished ACL facet prominence, squaring of the lateral and medial tibial plateaus, and broadening and flattening of the tibial spine. Employing this strategy with clinical MRI in further large-scale analyses could facilitate the identification of additional 3D morphologies as well as the injury risk, operative outcomes, and joint degeneration.
